# From facing fears to experimental frontiers: immersive cinematic virtual reality environments as a catalyst for innovation in behavioral neuroscience

**DOI:** 10.3389/fpsyt.2026.1893646

**Published:** 2026-09-07

**Authors:** Shaira Berg, Paul C. Fletcher, Trevor W. Robbins, Amy L. Milton

**Affiliations:** 1 Department of Psychology, University of Cambridge, Cambridge, United Kingdom; 2 Department of Psychiatry and Behavioral Sciences, University of Texas at Austin, Austin, TX, United States; 3 Department of Psychiatry, University of Cambridge, Cambridge, United Kingdom

**Keywords:** behavioral neuroscience, cognitive neuroscience, experimental design, immersive neuroscience, learning, maladaptive behavior, psychiatry, virtual reality

## Abstract

Virtual reality (VR) has gained traction in psychiatric research in exposure-based interventions for phobias, simulations of hallucinations in psychosis, and adaptations of cognitive-behavioral therapy. These applications demonstrate that emotionally immersive digital environments can meaningfully engage pathological processes in controlled settings. Yet VR’s potential in psychiatry goes beyond providing new contexts for well-established therapeutic interventions. Here, we argue that while emotionally rich, interactive VR scenarios can be leveraged to radically improve translational experimental design in behavioral neuroscience, there are not yet clear conceptual frameworks for the incorporation of these elements. Traditional lab tasks with theoretical implications for therapeutic interventions often rely on abstract procedures that fail to capture the immersive, affective dynamics of real-world maladaptive behaviors. Here, we discuss how high-fidelity VR scenarios simulate complex emotional naturalistic contexts while retaining precise experimental control. We illustrate this drawing on our recent work using high-fidelity, game-engine-based VR scenarios and consider how these environments offer a scalable and accessible platform for large-sample studies, as VR hardware becomes more common in the household. We propose a set of dimensions called the Immersive Behavioral Translation Framework (IBTF) that suggest conceptual considerations for VR in neuroscientific research: (i) The controllability of immersive environments; (ii) features of immersive environments that bridge ecological validity gaps; and (iii) the relatability of these emotionally immersive experiments to clinical conditions in translational research. We illustrate this framework with reference to emerging findings in associative learning and threat sensitivity, while emphasizing the broader implications for rethinking how we study cognition, emotion, and psychopathology.

## 1 Introduction

Much research in psychiatry draws on highly validated theoretical models such as learning theory, reinforcement learning, and threat sensitivity to explain the mechanisms underpinning psychopathology (1–3). These theoretical models help to understand psychopathology through cognitive and behavioral processes, often attempting to link them to underlying neural mechanisms through neuroimaging techniques (4, 5). Understanding these processes, and how they may be disrupted, facilitates the development and testing of therapeutic interventions for conditions such as schizophrenia, obsessive-compulsive disorder (OCD), and anxiety disorders (6, 7). These experimental procedures, however, are frequently forced to sacrifice ecological validity for precise experimental control and this can limit their translational potential. In this respect, virtual reality (VR) offers an exciting opportunity to refine and ecologically validate these approaches, helping to bridge the gap between lab-based theory and real-world maladaptive behavior. This will help to better understand how such behavior arises and persists as well as how it may respond to different interventions (8, 9).

When considering the use of VR in neuroscientific research, it is important to distinguish between the different types of immersive technologies available in our ever-evolving digital world. The key types of VR are: traditional VR, augmented reality (AR), mixed reality (MR) and serious games (9–12). Traditional VR refers to the technology that fully simulates a person’s three-dimensional (3D) environment in a computer-generated space, while AR refers to an integration of the virtual world anchored to concrete objects or locations present in a person’s perceived physical environment (9–11). MR on the other hand, is a mix of these two technologies that blends both physical and virtual worlds (10, 12). Serious games refer to digital games that are created with both entertainment value and at least one additional goal such as learning or health-related goals which can be applied in education and in clinical practice (10, 12). As VR technology progresses, AR and MR technologies will become more mainstream due to the ability to integrate both physical and virtual worlds with one another. For the purpose of this perspective piece, we focus on the current and future use of traditional, fully immersive VR environments specifically and consider the scope of combining serious games with research in behavioral neuroscience.

The use of VR in neuroscience and psychiatry has already had a positive impact on research and treatment (13–15). In psychiatry, VR is most often used for exposure therapy interventions in anxiety disorders, phobias, post-traumatic stress disorder (PTSD), and some types of OCD (16–20). In phobia and PTSD research, participants are confronted with the trigger or simulation of the traumatic experience that causes distress in a controlled and safe environment, while retaining a compelling sense of presence and immersion, thus allowing patients to reframe and attribute new meanings and associations to the experience (14, 19–23). VR is similarly utilized in psychosis to simulate and understand psychotic experiences and capture the associated impacts of experiencing hallucinations (24, 25). Cognitive rehabilitation also leverages VR technologies, for example in training executive function and working memory and through integration with cognitive behavioral therapy, including for social anxiety, where participants are exposed to triggers in a structured therapeutic manner (26–28).

While these applications are therapeutically beneficial, they are often used from a symptom-triggering standpoint (22). However, VR can also be used to increase the immersive nature of existing, validated behavioral procedures that allow insight into perturbed cognitive mechanisms under naturalistic conditions to inspire and refine future clinical interventions (8, 9). Emotionally immersive VR refers to VR experiences that can safely evoke a specific emotion or feeling in the participant, such as VR scenarios that include phobia-specific stimuli with emotional-attributions for the participants (i.e. fear; 21). As emotionally immersive VR already has therapeutic applications and has been shown to work in inducing and reducing symptomology in many conditions such as phobias, contamination OCD, and schizophrenia (21, 27, 28) using these techniques in experimental psychiatry has great potential in transforming the understanding of human behavior (8, 9). However, these applications have not yet been well-defined.

As VR has already been implemented in current behavioral research and is becoming more widespread for enhancing real-world generalizability, this justifies the proposal of a set of dimensions through which VR could improve experimental work in cognitive neuroscience through a series of potential conceptual scaffolds. These considerations could enable scientists to aid the innovation and improvement of the design of highly immersive behavioral tasks along three key dimensions of (i) Controllability; (ii) Ecological Validity; (iii) Translational Interpretability, that we propose as the Immersive Behavioral Translation Framework (IBTF). The current paper will illustrate the key concepts underpinning the IBTF using the VR neuroscience literature, rather than provide a comprehensive systematic overview of the field (for a review of the use of VR in clinical and behavioral psychology please see: 13, 22, 29, 30).

This will help scientists to think along these dimensions around designing behavioral experiments using high-fidelity immersive VR techniques and guide the design and interpretation of such tasks, to support a shared language of interdisciplinary collaboration across the behavioral neurosciences, psychiatric research and the entertainment industry.

- Controllability of Immersive Environments: firstly, while lab-based experiments must be highly controllable, these considerations can be extended to experiments with immersive nature and cinematic realism. While open-world, exploration-based experiments can successfully exist in VR to better understand individual variations in behavior (e.g. in decision making), it is important that experiments still consider implementing and embedding features from validated procedures with scientific rigour, maintaining control over aspects such as stimulus type, timing, structure, and relevance to the hypothesis being tested where possible. This poses the explicit procedural question: *can the variables be adequately isolated and manipulated?*
- Ecological Validity: next, while lab-based experiments should have high levels of ecological validity as the task should closely resemble the behavior being measured or captured to evoke naturalistic attention, a careful balance between realistic scenarios and fictional, hypothetical experiences can improve the richness of findings. These processes can be captured despite fictional, unlikely contexts and may even help to measure behavior in a way that is sensitive and avoids direct symptom provocation in psychiatric experiments. For example, including space or monsters to elicit fear will allow naturalistic threat-response behavior to be evoked despite the fictional context, avoiding directly triggering or upsetting a participant with, for example, severe OCD related to a real-world stressor. Therefore, experimenters must ask themselves: *to which level does the task approximate and capture naturalistic behavior?*
- Translational Interpretability: finally, these experiments can attempt to bridge the gap between tightly controlled lab experiments, and the emotional richness of every-day lived experience to simulate transdiagnostic experiences that contribute to the development and persistence of clinical conditions. Such experiments would therefore improve the validity and accuracy of Phase II clinical trials and increase translatability to Phase III and psychiatric intervention. Here, the question should be posed: *how does performance map onto the development of real-world psychiatric intervention?*

## 2 Virtual reality in current behavioral research

Traditional behavioral research relies heavily upon abstract, computer-based tasks to study learning, memory, emotion, and fear conditioning, where participants respond to stimuli on a two-dimensional (2D) screen (31–33). It is assumed that the challenges posed by these tasks, and the ensuing responses, can be generalized to naturally occurring behavior in the real world and thereby are key to understanding psychiatric conditions and their interventions. However, it remains unclear the degree to which such abstract interactions can truly model how participants perceive and respond to environmental cues in everyday life. These tasks typically lack narrative immersion and may result in low participant engagement, potentially compromising data quality and generalizability. While controllable, this poses the question of how similarity to real-world behavior can be improved along the ecological validity arm of the IBTF. Moreover, to increase comparability to non-human animal studies, hyper-immersive virtual reality studies could increase the perceived ‘inescapability’ of the task similar to ones observed in animals. Previous reviews have highlighted how the role of informed consent and the right to withdraw in human studies turns these ‘inescapable’ stress scenarios in pavlovian fear conditioning into ‘escapable’ stress, resulting in difficulty implying translatability to the animal procedures (34). These considerations could additionally probe the translational interpretability query of the IBTF.

There is an increasing tendency for researchers to improve engagement and motivation by ‘gamifying’ tasks through the addition of points, audio-visual adornments and a simple narrative, but this too has limitations (35, 36). Tasks that are designed to induce fear, threat, and stress particularly benefit from ecological innovation. Commonly used state-stress methods include cold pressor tests, the Trier Social Stress Test and the use of aversive sounds, videos, and imagery (37–43). These methods rely on delivering stress that happens to the participant in ‘real-life’ during the experiment; therefore, utilizing VR to immerse participants in threatening scenarios has the advantage of engaging participants in realistic narrative environments similar to physical stress tasks (44–48).

Moreover, tasks that are utilized in neuropsychological cognitive assessment could additionally be improved using VR technologies, for example in decision-making and multi-tasking assessments (49, 50). A real-life shopping scenario was introduced in order to capture deficits in executive function in patients with frontal lobe injuries, which allowed them to navigate an emotionally familiar environment and make decisions (51). In cognitive aging research, older adults seem to function competently in complex everyday scenarios despite deficits on cognitive tasks in the laboratory, suggesting that more realistic cognitive experimental procedures may better capture naturally occurring behavior and function (52).

Moreover, VR has additional translational implications beyond symptom-provocation and clinical assessment in the clinical literature (22, 25, 28, 53, 54), with recent studies presenting the potential of immersive environments in supporting cognitive and social functioning, for example by simulating environments where participants can practice skills that can improve their day-to-day lives (53, 55–58). This provides evidence of VR being valuable beyond the innovation of behavioral procedures to probe cognitive mechanisms in the lab, as VR has the potential to transform the interventions for rehabilitation in individuals experiencing psychiatric conditions.

Furthermore, VR provides an opportunity to simulate naturalistic contexts through multi-sensory stimuli including both auditory and visual landscapes to enable more realistic simulations of real-world responses (40, 59). VR also provides high levels of environmental control across both visual and auditory modalities (60, 61) and it has been found that behavioral realism improves the perceived immersion of the experience (62). These multi-sensory environments can be designed to evoke naturalistic emotional reactions while allowing researchers to track behavior in response to specific parameters, particularly in the context of threat sensitivity and aversive learning (63, 64). VR testing has also previously been utilized in cognitive neuroscience to measure human navigation, which could additionally improve the richness of emotional testing including geospatial dimensions, providing the possibility to replicate engagement with one’s surroundings in a realistic spatial manner (65).

## 3 Using high-fidelity VR environments to capture theoretical phenomena

The efficacy of VR stress induction procedures is typically validated using a combination of physiological and self-report measures, providing evidence that VR stressors are comparable to real-life physical stressors, however VR allows more variation in procedural design in terms of the kinds of environments and immersion. Galvanic skin response (66, 67), pupillary dilation (68, 69), and cardiovascular measures such as heart rate and heart rate variability (HRV) (70–72) provide objective markers of arousal that can be implemented in VR environments. HRV captures adaptability to environmental stressors and is highly correlated with mental and physical health and has additionally already been utilized in VR experiments (45, 73). This supports the use of such physiological measures together with VR environments to capture and evoke state-stress in ways that are ecologically valid, emotionally salient, ethically justifiable and theoretically grounded, offering a new foundation for experiments in behavioral neuroscience.

Our collaboration with multi-BAFTA award-winning video games studio Ninja Theory has shown that highly cinematic, immersive horror environments are effective in evoking physiological and psychological stress responses as measured by heart rate measures and salivary alpha-amylase (45, 46, 74, 84). Our work has shown that such environments not only have a high level of lab-based controllability; they also offer the opportunity to exercise the same level of stimulus precision as traditional procedures, with the added advantage of contextual realism. If we can build emotionally realistic, controllable models of behavior and how this behavior becomes dysfunctional, we may be able to mimic an environment where we are more likely to capture naturally occurring patterns of behavior. This is particularly true when the environment has a storyline as participants are more likely to be immersed and rely on more instinctive pathways in these environments (45, 75, 76).

Other theoretical phenomena such as latent inhibition in schizophrenia (LI) and learned helplessness (LH) in depression can also be made more emotionally immersive through virtual reality techniques (77, 78). Patients who are acutely hospitalized with schizophrenia exhibit LI deficits with difficulty filtering out irrelevant stimuli, which can predict learning patterns that may help to identify predispositions for the condition (79). Moreover, LH in depression involves tasks where participants are unable to escape an aversive outcome no matter the responses or strategy changes they make (77, 78). Currently, such tasks are presented on a 2D computer screen where participants are tasked with making responses in an abstract procedure lacking ecological validity (i.e. pairing fractal images with key presses; 77, 78). Making these procedures more immersive could involve presenting the CSs and environment where CRs are recorded in a more immersive storyline-based manner, that could better capture the unconscious learning patterns that such individuals exhibit in the real world. The greatest advantage of utilizing VR to study these conditions is that one is able to create and simulate multiple different environments with the same basic kit, including multi-sensory stimuli through the headset and headphones and physiological recording devices. This widens the possibility of multiple variations of a procedure to study these behavioral phenomena that can be generated at low cost.

## 4 The IBTF in practice

To demonstrate how the IBTF can be effectively implemented in behavioral neuroscience, we examine two examples of recent work by Rodriguez Lopez et al. (48) in comparison with 2D experiment by Tzovara et al. (80) and our recent work Berg et al. (74, 81) in comparison with 2D experiment by Quail et al. (82). Tzovara et al. (80) conducted a fear conditioning experiment in humans, with the use of a 2D computer task where participants were instructed to associate various abstract cues with aversive electric shocks while measuring galvanic skin response and pupillary dilation. This type of computer-based task is well validated in eliciting threat-related behavior and measuring physiological outcomes associated with fear behavior, but lacks extrapolation to real-life scenarios where threat-related behavior may be evoked due to its abstract nature. Rodriguez Lopez et al. (48) presented a fear conditioning experiment using a VR human avoidance learning task where participants were placed in a room with various 3D shapes and aversive sounds.

Moreover, Quail et al. (82) presented a classic pavlovian-instrumental transfer (PIT) task involving a 2D vending machine and various colored cues predicting snack rewards, which has also been well validated in eliciting PIT effects across many replications (e.g. 83) Berg et al. (84) presented an aversive pavlovian-instrumental transfer conditioning task, implemented in VR using the IBTF in addition to the use of cinematic threat induction scenarios. Here, the abstract task was replaced with a fictional zombie game where participants were asked to learn different associations between patterns and pills to cure them from a fictional zombie virus, while alpha-amylase and heart rate were additionally measured. The IBTF can therefore be utilized to adapt a behavioral task to increase immersion and realism.

For the first aspect of controllability, the basic conditioning task structure is kept the same as the 2D task counterparts (e.g. Quail et al. (82) for a PIT task and Tzovara et al. (80) for a fear conditioning task), where cues are presented for the validated durations and in the validated structure associated with the learning effects and evoking this in the lab. Controllability is essential despite the storyline and additional factors contributing to immersion being implemented in the task. Moreover, the Berg et al. (84) study carefully controlled the environment, where the compound threat conditions were curated through the use of ambient sound and threat evoking scents that are kept constant for each condition across all participants and Rodriguez Lopez et al. (48) used a basic gray room with nothing but a pedestal for the CS shapes to appear on and a lightbulb in the space. Physiological measures were also implemented in a controlled manner in Berg et al. (81), utilizing protocols from previous biomarker designs where heart rate was measured through the use of a pulse finger monitor on a hand that was kept still throughout the entire task, despite the immersive nature.

For the second aspect of ecological validity, an experimenter must question how to best evoke threatening behavior in a scenario that can elicit natural real-world behavior, while remaining sensitive to potential symptom triggers. The examples by Quail et al. (82) and Tzovara et al. (80) relied on abstract cues (different colored squares) which results in an experiment that lacks ecological validity and immersion. To elevate this type of experiment, our example of using a fictional ‘zombie scenario’ allows emotions such as urgency to avoid threat and fear of unpleasant outcomes to be evoked, while maintaining an element of distance from real-world triggers (84). A focus group of individuals with diagnosed OCD and contamination fears was administered prior to the experiment in order to validate the storyline and the various scenario stimuli used in the task, as the purpose of this experiment was to evoke contamination threat throughout the experiment. Moreover, in the procedure presented in Rodriguez Lopez et al. (48), the use of a basic gray room, with 3D shapes serving as the CS stimuli, allowed the task to be presented in an immersive 3D world without manipulating additional extraneous variables, providing evidence that translating learning tasks to immersive environments does not always require an additional fictional storyline.

Finally, when considering translatability, conditioning tasks such as the PIT and fear conditioning task have been validated to better understand learning and maladaptive behavior in psychopathology. The storyline immersive context introduced in Berg et al. (81) allows a way to simulate the general and specific learning pathways that may be implicated in OCD i.e. engaging in a generalized repetitive or maladaptive behavior to self-soothe and avoid an unpleasant overall outcome; an example would be repeatedly turning a light switch on and off to avoid a feared death in their family. Moreover, the 3D room presented in Rodriguez Lopez et al. (48) allows translatability to rodent designs where the animal is physically presented various cues in a basic box without additional distractors. Therefore, the immersive tasks presented increase the translatability of an abstract learning task to one that better simulates both cross-species and clinical phenomena and can be used to inform therapeutic interventions.

## 5 Discussion

The VR headset is becoming more commonplace in the household, with a global cumulative estimate of over 26 million actively used VR headsets by the end of 2023 alone (85). This could allow online psychological experiments to also use VR approaches, giving rise to the potential for enormous datasets that could help understand the continuum of transdiagnostic behavior, relevant to psychiatric conditions present in the general population. In 2022, the streaming platform Netflix released an interactive film titled ‘Bandersnatch’ as part of the popular ‘Black Mirror’ series. This film garnered 45 million viewers in the first month with 94% of participants making interactive choices, as they were able to navigate the film by selecting different options along a decision-making tree (86). Future VR experiments in collaboration with gaming and production studios could potentially follow similar structures, with enhanced yet embedded validated lab-based tasks, in which participants can consent in the comfort of their own homes to take part in immersive environments that capture a rich wealth of data on learning, emotion, memory, and the development of maladaptive behaviors while considering the various aspects of the proposed IBTF. Such collaborations could also move into the ‘serious games’ space, by providing structured ‘experimental experiences’ or ‘games’ that are available to the general public, which combine entertainment and experimental data collection, potentially widening the scope of such experiments beyond the lab. While ambitious, garnering cinematic structures for psychological experimentation may hold the future of behavioral neuroscience and innovation of such methodology.

Despite the potential of these immersive VR environments to expand the scope of experiments in behavioral and cognitive neuroscience, there are several limitations of the use of such equipment that are important to consider. Firstly, the current lack of conceptual framework for how immersive VR should best be integrated into behavioral experiments poses the risk for such tasks to lack replicability and be difficult to compare across different groups. Therefore, such tasks could continue to adhere to a certain level of experimental control and implement cognitive theory through validated skeletal task designs (i.e. the LH or LI human experimental procedure (77, 79)) including naturalistic immersion carefully as to not contaminate these procedures to the point where it is difficult to isolate specific cognitive and behavioral mechanisms. Moreover, the methodological and experimental methods used to create cinematic, story-driven VR experiments may be prone to differences in software, equipment, and narrative design that affects the style of task, therefore communication and pre-registration techniques are important to ensure cross-lab replication.

Furthermore, it is important to note that while immersive VR environments offer a strong advantage in terms of realism and emotional engagement, traditional experiments in behavioral neuroscience continue to offer key benefits due to their experimental control and explicit isolation of specific cognitive processes. Due to this, it is important to note that VR experiments should be implemented as a second step to traditional 2D tasks, as the validation and development of 2D tasks investigating processes such as PIT is essential to ensure that the correct behavioral mechanisms are being measured before extrapolating it to a more immersive, 3D design. Therefore, researchers should keep in mind that the further development and testing of VR tasks do not replace the need for the use of 2D designs as these offer the foundation for more immersive, engaging tasks further down the line.

There are several specific preventative measures that can improve replicability. For example, labs can pre-register their task on open-source platforms such as Open Science Framework to share hypotheses, exclusion and inclusion criteria, task structure and stimulus timings, outcomes and analysis pipelines. The VR experiments and scenarios can additionally be shared and uploaded to be used across labs and experiments investigating similar hypotheses. Moreover, hardware specifications can be explicitly shared in experimental methods sections, and experiments can be hosted on platforms that are accessible from most modern VR headsets (i.e. screen mirroring an experiment in PsychoPy into any headset and uploading scenarios to a YouTube link). Experimenters should also share calibration procedures and could suggest alternative lower-cost implementation options if the experiment has been conducted in collaboration with a studio (i.e. sharing experiment stimuli and options for replicating the stimuli using accessible VR cameras). It is also important to note that the implementation of home-based online VR experiments, especially with immersive environments, is likely to generate additional challenges than those observed in online experiments more generally (87). The absence of direct clinical or experimental supervision could result in implications for participant safety in terms of cybersickness and adverse effects and could result in the variability of data quality due to differences in available hardware. There may also be privacy concerns due to the lack of control over the environment in which the experiment takes place. Due to this, researchers should consider implementing at-home experiments by for example recruiting participants with the same hardware, and being present over a video-call to ensure that they are able to help the participant and send aid should there be any adverse effects of note.

It is also important to consider some of the more explicit clinical ethical limitations as such VR experiments are useful in simulating phobias and psychosis-like experiences in clinical populations to better understand the cognitive and behavioral pathways. Symptom provocation and exacerbation are vital to mitigate as best as possible through careful monitoring and safety mechanisms such as strict informed consent and contingency plans if a participant cannot tolerate a scenario. This is due to both psychological and physiological issues, including cybersickness and heightened anxiety particularly when they involve threatening stimuli (11). Moreover, scenarios that receive ethical approval for human research could be explicitly made open-source under this premise to further validate the scenarios as being safe and not harming the participant either psychologically or physically through motion sickness or similar, which could also be useful for better replication and safety management of at-home online-based experiments.

The risks of cybersickness and emotional distress that could exacerbate symptoms can additionally be mitigated through safeguarding techniques, including extensive clinical screening, and exclusion and inclusion criteria carefully monitoring for factors such as specific phobias to experiment stimuli. Moreover, a safety protocol is essential to be implemented for cybersickness and adverse effects where the participant is able to withdraw from the procedure at any time and is monitored throughout the experiment for any distress with contingency plans in place for additional medical help if necessary. Most importantly, the immersive, high-fidelity environments do not need to recreate a participant’s real-world symptomology; instead, fictional scenarios that have high levels of engagement can allow investigation into behavioral mechanisms underpinning psychopathology without directly triggering symptoms in a vulnerable participant. These immersive environments furthermore allow controllability of intensity to ensure safety during stress and fear induction in the lab.

Another key consideration is the role of individual differences in digital literacy and gaming experience, as there may be wide variation across participants, which is essential to consider in terms of implementing the IBTF. With the rise of young people engaging in video game use (88, 89), the participant population has increasingly higher levels of media literacy and visuospatial experience in immersive environments due to the so called ‘technological boom’. Therefore, controlling for this variability through including explicit covariates or dividing groups based on video game usage and exposure can further help the controllability axis of the IBTF. Moreover, the individual differences in vestibular balance and postural sway are also important to consider and can be mitigated by procedural decisions such as having the participant seated for the duration of the experiment (90–92). Such procedural decisions should be well-documented and preregistered to further enhance the rigor and replicability of immersive environments as experimenters innovate these experimental designs.

Finally, hyper-immersive VR scenarios currently require close collaboration with game studios, however this expertise is difficult to access particularly for small labs without a collaboration set up. However, we suggest that with the way in which creating personalized VR experiences is becoming more accessible through game design platforms such as Unity (93), as technology continues to advance, labs will likely be able to design and access VR scenarios that suit the needs of a particular experiment themselves. Moreover, they are also able to use scenarios that are open-source and already available for researchers to implement in their experimental design as Ninja Theory is currently in the process of doing. Furthermore, the implementation of the IBTF does not necessarily require bespoke cinematic environments developed by large movie and game studios; rather, the framework is designed to be applicable across many platforms and does not rely on production value for successful application. While cinematic realism can help, there is evidence that it is not necessary as long as the basic sense of presence is fulfilled, which relies more on context and psychological factors than high-resolution graphics (23, 94, 95). Thus, low-cost VR video cameras could be used to capture real-world environments, rather than relying on specifically designed environments by graphics designers, to achieve essentially the same goals and ‘cinematic realism’ of highly funded studies with studio backing.

In conclusion, to better understand behavior that contributes to psychiatric conditions and maladaptive transdiagnostic traits, innovative experimental procedures that can capture the emotional unpredictability, intensity and complexity of real-life experiences are warranted. These environments have the potential to model real-world behavior in controlled and replicable ways, however require a conceptual framework for consistent implementation suggested by the proposed IBTF model. The integration of cinematic-like game design with well-established and validated cognitive tasks allows the potential to explore threat, stress, and arousal in novel ways, better understanding how they interact with decision-making, memory, and learning in everyday life. Thus, carefully considered emotionally immersive VR techniques are a necessary addition to current experimental design to better mirror the human experience in the lab. This potential requires close collaboration between both the scientific and creative industries and the willingness to rethink how emotional and behavioral experiments should be conducted in neuroscientific research and beyond.

## Data Availability

The original contributions presented in the study are included in the article/supplementary material. Further inquiries can be directed to the corresponding author.
